# From Bowen disease to cutaneous squamous cell carcinoma: eight markers were verified from transcriptomic and proteomic analyses

**DOI:** 10.1186/s12967-022-03622-1

**Published:** 2022-09-09

**Authors:** Tang Biao, He Cai-feng, Lu Xiao-hong, Chang Xiao-li, Liu Wen-bei, Wang Jun, Ci Chao, Yuan Tao

**Affiliations:** Department of Dermatology, Yijishan Hospital, the First Affiliated Hospital of Wannan Medical College, Wuhu, Anhui China

**Keywords:** Cutaneous squamous cell carcinoma, Bowen disease, Proteomics, Biomarkers

## Abstract

**Background:**

Bowen's disease is a cutaneous squamous cell carcinoma (CSCC) in situ. If left untreated, BD may progress to invasive CSCC. CSCC is one of the most common cutaneous carcinoma in the elderly and the advanced, metastasis CSCC usually have a poor outcomes. However, the mechanisms of invasion and metastasis from Bowen’s disease to CSCC is complicated and still unclear.

**Objectives:**

The aim of this study was to explore the biomarkers and molecular alterations in Bowen’s disease development process via analyzing the proteomics changes in tissues of CSCC, Bowen disease and healthy skin.

**Methods:**

A total of 7 individuals with CSCC (5 for proteomics study and 2 for validation), 7 individuals with Bowen disease (5 for proteomics study and 2 for validation) and 7 healthy controls (5 for proteomics study and 2 for validation) presented to the Department of Dermatology, Yijishan Hospital, the First Affiliated Hospital of Wannan Medical College between January 2021 and December 2021 were enrolled. The proteomics analysis was performed to screen differentially expressed proteins/gens (DEPs/DEGs) in the lesions of CSCC, Bowen disease and healthy skin tissues. The transcriptomic data (GSE32628) of CSCC was selected and downloaded from the GEO database. The common DEGs in our proteomics results and GSE32628 between CSCC and healthy skin tissues were selected. And then, the common DEGs which significantly up or down-regulated between CSCC and Bowen disease in our proteomics results were further screened to identify using Western blot methods in the validation group. CSCC A431 cells were transfected with SERPINB1 small interfering RNA (si-SERPINB1) or small interfering RNA negative control (si-NC). To explore the effect of SERPINB1 silencing on migration and invasion ability of A431 cells.

**Results:**

A total of 501 proteins were differentially expressed between the CSCC and healthy skin tissues, with 332 up-regulated and 169 down-regulated at least 1.5-fold with a *P* value < 0.05. These DEPs involved multiple biological functions such as protein binding process, immune, inflammation, ribosome, protein digestion and absorption, ECM-receptor interaction, focal adhesion, PI3K-Akt signaling pathway and others. A total of 20 common DEGs (COL3A1, LUM, TNC, COL1A1, ALDH3A2, FSCN1, SERPINB4, SERPINB1, CD36, COL4A1, CSTB, GPX3, S100A7, ACTN1, SERPINB3, S100A8, RAB31, STAT1, SPRR1B, S100A9) between CSCC and healthy skin tissues in GSE32628 and our proteomics results were found. Besides, the proteins of TNC, FSCN1, SERPINB1, ACTN1 and RAB31 in CSCC were significantly up-regulated, while COL3A1, COL1A1 and CD36 were significantly down-regulated relative to Bowen disease in proteomics results. These proteins were mainly involved in multiple pathways, including Focal adhesion, ECM-receptor interaction, Human papillomavirus infection, PI3K-Akt signaling pathway, PPAR signaling pathway, AMPK signaling pathway and others. These eight proteins were selected for further validation. According to the Western blotting analysis, when compared with the Bowen disease and healthy skin tissues, we found that the relative expression levels of TNC, FSCN1, SERPINB1, ACTN1 and RAB31 in the CSCC were significantly increased, while COL1A1 and CD36 were significantly decreased, and the differences were statistically significant (*P* < 0.05). Furthermore, the relative expression levels of TNC, FSCN1, SERPINB1 in the Bowen disease were also significantly increased, while the COL3A1 were also significantly decreased relative to the healthy control. SERPINB1 siRNA inhibited the expression of SERPINB1 at mRNA and protein levels in the A431 cells. After interfering with the expression of SERPINB1, the migration and invasion ability in the A431 cells were significantly decreased (*P* < 0.05).

**Conclusions:**

This study highlights that eight proteins, TNC, FSCN1, SERPINB1, ACTN1, RAB31, COL3A1, COL1A1, CD36, were significantly associated with the mechanisms of invasion and metastasis in Bowen’s disease.

**Supplementary Information:**

The online version contains supplementary material available at 10.1186/s12967-022-03622-1.

## Introduction

Cutaneous squamous cell carcinoma (CSCC) is one of the most common cutaneous carcinoma in the elderly. With the extension of life expectancy in human, the prevalence of CSCC keeps rasing, with an annual global incidence more than 1 million [1]. CSCC in situ is also known as Bowen’s disease (BD). If left untreated, BD may progress to invasive CSCC with the incidence of 3% ~ 5% [2]. Photodynamic therapy is an alternative to surgery for BD. However, a retrospective study demonstrated that the 16 patients with BD who developed one or more CSCC after Photodynamic therapy treatment [3]. The exact mechanisms by which BD progresses to CSCC are complicated and merits more attention. In addition, the patients with CSCC easy to occur metastasis and with a poor prognosis. Genders RE et al. [4] published an excellent study regarding approximately 90% of CSCC metastases appear within 2 years after the initial diagnosis.

The proteomics technology has been successfully used to identify DEPs/DEGs in tumor tissues and to provide insights into the molecular mechanisms underlying and/or potential therapy targets for these complicated diseases [5]. The proteomics changes in tumor tissues can directly and veritably reflect the local tumor microenvironment, thus providing a way to investigate the molecular mechanisms of tumor. Formalin-fixed paraffin-embedded (FFPE) tissue blocks represent a valuable source of samples for clinical research, as pathology departments routinely archive them in vast numbers. According to previous studies reported that FFPE tissues could provides opportunity for retrospective mass spectrometry-based (MS) proteomic profiling [6, 7]. Bowen disease, also called cutaneous squamous cell carcinoma in situ, has been described as a premalignant intraepidermal lesion of the skin. Better understanding of aggressive growth and/or metastatic mechanisms of CSCC is urgent. Therefore, in this study, we applied this proteomics method to explore the proteomics change between CSCC, Bowen disease and healthy skin tissues. And next, we aim to explore some significant target proteins associated with CSCC and further verification by Western blot method.

## Materials and methods

### Patients

A total of 5 inpatients with CSCC (n = 5) and 5 inpatients with Bowen disease (n = 5) between January 2021 and December 2021 were selected in this study according to the postoperative histopathology results. On the other hand, 5 inpatients with pigmented naevi were defined with healthy controls. The CSCC tissues, Bowen disease tissues and healthy skin tissues (more than 2 cm apart from pigmented naevi) were obtained after surgical resection.

The 15 enrolled individuals comprised 8 men and 7 women with a male: female ratio of 8:7. The age ranged from 52 to 87 years with a mean age of (67.13 ± 9.20) years. The main lesion site was nose (9, 60%) followed by cheek (6, 40%). There was no significant differences in age, gender and tissues location between the three groups. The clinical characteristics of all individuals are shown in Table 1. The TNM staging of patients with CSCC at greater risk of metastases were identified by the literature’s reported [8]. The archival formalin-fixed paraffin embedded (FFPE) of CSCC, Bowen disease and pigmented naevi-adjacent normal skin tissues were obtained from the Department of Pathology in our hospital and for further proteomics study. All individuals provided written informed consent for the inclusion of their FFPE samples in the present study. The present study was approved by the Ethics Committee of Yijishan Hospital, the first affiliated hospital of Wannan Medical College.

**Table 1:** The clinical characteristics of 15 enrolled individuals

Groups	Case	Age, years	Gender	Tissue locations	TNM staging
CSCC (n = 5)	Case 1	67	Male	Cheek	pT2 pN1 Mx
	Case 2	71	Male	Nose	pT2 pN1 M1
	Case 3	69	Male	Nose	pT1 pNx M1
	Case 4	80	Female	Cheek	pT3 pN1 M0
	Case 5	87	Female	Nose	pT3 pN1 Mx
Bowen disease (n = 5)	Case 1	68	Male	Cheek	Primary CSCC
	Case 2	73	Male	Nose	Primary CSCC
	Case 3	58	Female	Nose	Primary CSCC
	Case 4	75	Female	Nose	Primary CSCC
	Case 5	59	Male	Cheek	Primary CSCC
Healthy controls (n = 5)	Case 1	81	Female	Nose	—
	Case 2	64	Male	Nose	—
	Case 3	74	Female	Cheek	—
	Case 4	68	Female	Nose	—
	Case 5	65	Male	Cheek	—
Statistics value	—	1.382	0.536	0.000	—
*P* value	—	0.288	0.765	1.000	—

The “—” indicated no significant differences

### Proteomics study and Bioinformatics analysis

#### Proteomics study

Protein Extraction: Microsections of CSCC (n = 5), Bowen disease (n = 5) and healthy controls (n = 5) from the Department of Pathology in our hospital were collected. Proteins were extracted from the microdissected tissue samples and dewaxed using the method which described previously [9]. Trypsin Digestion: About one volume of pre-cooled acetone was added in the same protein concentrations of each sample and mixed. And then four volumes of pre-cooled acetone was added in the each protein sample and precipitated at − 20 °C for 2 h. After centrifugation at 4500*g* for 5 min at 4 °C, the supernatant were discarded. We collected the sediment and washed with pre-cooled acetone for three times. The protein sample was then diluted by adding 200 mM TEAB and trypsin was added at a 1:50 trypsin-to-protein mass ratio for the first digestion overnight. The dithiothreitol (DTT) was added in each protein sample with a final concentration of 5 mM and stand still at 56 °C for 30 min. Then, iodoacetamide (IAA) was added to make the final concentration at 11 mM, and incubated at room temperature for 15 min in the dark. LC–MS/MS analysis: The tryptic peptides were dissolved in solvent A (0.1% formic acid and 2% acetonitrile) and separated using an NanoElute UHPLC system. The parameters were set as 6–24% solvent B (0.1% formic acid and 90% acetonitrile) for 70 min, 24–32% for 14 min, 32–80% for 3 min and 80% for 3 min at 450 nL/min flow rate. The peptides were separated by UHPLC system and injected into Capillary ion source for ionization and then analyzed by timsTOFTM Pro mass spectrometer.

### Bioinformatics analysis

Bioinformatics analysis mainly covers three domains: Gene Ontology (GO), Kyoto Encyclopedia Genes and Genomes (KEGG) and protein–protein interaction (PPI). The DEPs between CSCC, Bowen disease and healthy control were identified using a 1.5-fold change and the Wilcoxon test *P* < 0.05 threshold. Functional enrichment (GO and KEGG) were performed to identify the relationships among the target proteins associated with CSCC. The Universal Protein Resource (https://www.uniprot.org/, UniProt) is a well-known protein database, which consists of the UniProt knowledgebase (UniProtKB), the UniProt unique protein identifier archive (UniParc), and the UniProt reference sequence clusters (UniRef). Apart from protein sequence data, the UniProtKB has comprehensive annotations and is the core of the database [10]. Besides, the GO is the major bioinformatics technology used to unify the representation of gene and gene product attributes across all species. The GO annotation proteome was derived from the UniProt database and including cellular component, molecular function and biological process. The KEGG database was used to annotate protein pathways using the KEGG online service tool KAAS. The KEGG database enrichment analysis was used to identify enriched pathways from DEPs. A two-tailed Fisher’s exact test was used to test the GO and KEGG enrichment of the differentially expressed protein against all identified proteins, and a adjusted *P*-value < 0.05 was considered significant. On the other hand, the protein–protein interaction (PPI) network among DEPs was analyzed via STRING online software [11]. The Cytoscape (v.3.8.1) software was used to visualize the network.

### Database analysis

The transcriptomic data of CSCC (GSE32628) was selected and downloaded from the GEO database (https://www.ncbi.nlm.nih.gov/pmc/). In the GSE32628, a total of 13 samples with CSCC and paired 13 samples with normal tissues from 13 inpatients were selected and enrolled. RNA was isolated and gene expression profiles were obtained using HumanWG-6 v2 Expression BeadChips (Illumina). The transcriptomic data were calculated using GEO2R online (https://www.ncbi.nlm.nih.gov/geo/geo2r/). The differentially expressed transcriptomic data between CSCC and normal tissues were further identified using a log_2_FC > 2/ < -2 and adjusted *P* values < 0.01. Besides, in our proteomics study, we also identified DEPs between CSCC and healthy skin tissues using a 1.5-fold change and *P* values < 0.05. Next, a Venn diagram (http://bioinformatics.psb.ugent.be/webtools/Venn/) containing two lists of differentially expressed gens was drawn online to identify common gens that were significantly up- or down-regulated in CSCC. And then, the common gens which significantly up- or down-regulated between CSCC and Bowen disease were further filtered to verification using Western blot and Immunohistochemistry methods.

### Western blot

**Fig. 1:**
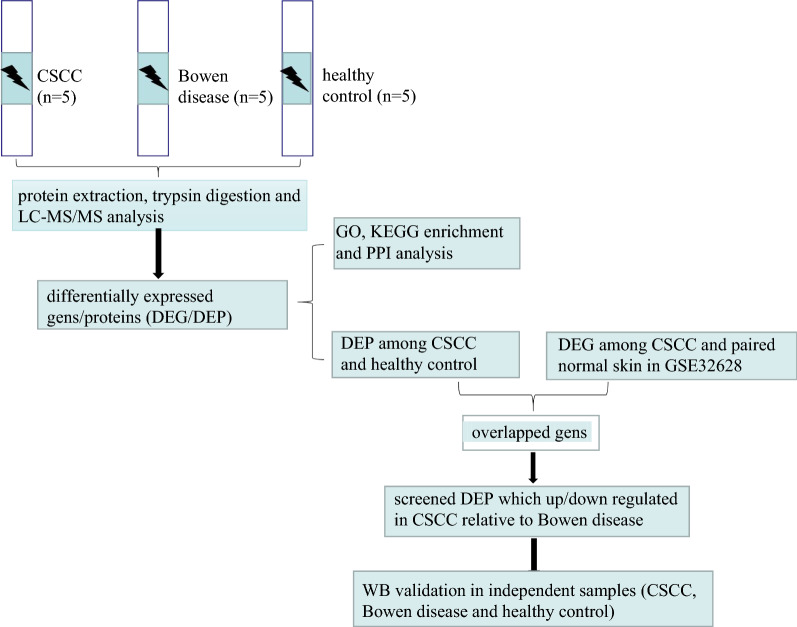
The experimental flow chart

The Western blot was used to verify the target proteins (TNC, FSCN1, SERPINB1, ACTN1, RAB31, COL3A1, COL1A1, CD36) expression levels in CSCC tissues relative to Bowen disease, healthy skin tissues. Besides, 2 inpatients with CSCC, 2 inpatients with Bowen disease and 2 inpatients with pigmented naevi were enrolled and defined as independent verification samples. The clinical characteristics of these 6 individuals are shown in Additional file 1: Table S1. The protocol of the western blotting assay was performed as follows. The CSCC (n = 2), Bowen disease (n = 2) and pigmented naevi-adjacent normal skin tissues (n = 2) were obtained after surgical resection. The tissues were lysed by RIPA lysis buffer (Beyotime Biotechnology, China) containing protease inhibitor cocktail (Beyotime Biotechnology, China) for 10 min on ice. Next, the lysates were then collected and transfer into new EP tubes and centrifuged at 8000*g* for 15 min at 4 ℃. The supernatant was collected for protein concentration quantified analyzed by BCA assay kits (Beyotime Biotechnology, China). Equal amounts of protein lysates (20 mg) were loaded into 4–15% precast SDSPAGE gels (Bio-Rad, Hercules, CA, USA) and then transferred to polyvinylidene fluoride membranes by the semi-dry transfer method. To block nonspecific protein binding, the membranes were incubated with 3% bovine serum albumin for 2 h. The membranes were washed with TBST for 3 min and then incubated with the corresponding primary antibodies overnight at 4 ℃. The membranes were washed with TBST for 3 min again and incubated with appropriate horseradish peroxidaseconjugated secondary antibodies for 2 h. They were washed again with TBST and visualized using enhanced chemiluminescence substrate kits (Beyotime Biotechnology, China) in a chemiluminescence imaging system (Bio-Rad, Hercules, CA, USA). The band intensity was quantified by Quantity One software (Bio-Rad, Hercules, CA, USA). The Western blot examination were performed using primary antibodies to TNC (HUABIO, Inc., China, catalogue number: ET1608-50, with antibody dilutions of 1: 500), FSCN1 (HUABIO, Inc., China, catalogue number: ET1705-18, with antibody dilutions of 1: 500), SERPINB1 (Abcam, Inc., USA, catalogue number: Ab181084, with antibody dilutions of 1: 2000), ACTN1 (HUABIO, Inc., China, catalogue number: EM1901-52, with antibody dilutions of 1: 500), RAB31 (Proteintech Group, Inc., China, catalogue number: 15485–1-AP, with antibody dilutions of 1: 800), COL3A1 (HUABIO, Inc., China, catalogue number: ER1906-50, with antibody dilutions of 1: 1000), COL1A1 (HUABIO, Inc., China, catalogue number: ET1609-68, with antibody dilutions of 1: 1000) and CD36 (HUABIO, Inc., China, catalogue number: ET1701-24, with antibody dilutions of 1: 500). The experimental flow chart are shown in Fig. 1.

### Effect of SERPINB1 silencing on migration and invasion of CSCC cell line

Cell culture: Two human CSCC cell lines (A431 and SCL-1) and a human immortalized keratinocytes cell line Hacat (as normal control) were obtained from American Tissue Culture Collection (Manassas, VA, USA). The cells were cultured in Dulbecco modified Eagle medium (DMEM) (Invitrogen, Thermo Fisher Scientific, Inc., Waltham, MA, USA) supplemented with 10% fetal bovine serum (FBS) (Invitrogen) and 1% penicillin/streptomycin (Invitrogen) and incubated at 37℃ in 5% carbon dioxide.

Real-time fluorescence-based quantitative PCR (RT-qPCR) and Western blot analysis were performed to detect the mRNA and protein of A431 and SCL-1 cells respectively, and then A431 cells with highly expressed SERPINB1 served as a research object (Additional file 6: Figure S3a and S3b). To evaluate effects of targeted silencing of SERPINB1 by small interference RNA on the migration and invasion of CSCC cell line A431. Cultured A431 cells were divided into 3 groups: experimental group transfected with SERPINB1 (si-SERPINB1-1 and si-SERPINB1-2), negative control group transfected with negative control (si-NC). After the RNA interference, the mRNA and protein of SERPINB1 in the above 3 groups were measured by RT-qPCR and Western blot analysis, respectively, and the effect of the RNA interference on the invasion and migration abilities of A431 cells was evaluated by Wound healing assay and Tran-swell. SERPINB1 knockdown: Two small interfering RNAs (siRNA1 and siRNA2) targeting SERPINB1 and negative control (si-NC) were designed and synthesized by GeneChem, Co., Ltd., (Shanghai, China). siRNAs were then transiently transfected into cells using Lipofectamine 2000 (Invitrogen). After 48 h culture, the transfected cells were further performed to next assays. The sequences were as follows: si-SERPINB1-1: 5ʹ-GCACTTGTGTCAAGGTCTTT-3ʹ, si-SERPINB1-2: 5ʹ-CTGTACAGAGTGGATCGTTT-3ʹ, si-NC: 5ʹ-TTCTCCGAACGTGTCACGTTT-3ʹ. The RT-qPCR and Western Blot assay were performed to evaluated the knockout efficiency of SERPINB1 in CSCC cell line A431. The RT-qPCR, Western Blot assay, wound scratch assays and Transwell experiment protocols were showed in Additional file material.

### Statistical analysis

Data are presented as the mean ± standard or frequency and were analyzed by student’s t-test, chi-square test using the SPSS software 20.0 (IBM Corp., Armonk, NY, USA). The differentially expressed microarray between two groups in GSE32628 were further identified using a log_2_FC > 2/ < -2 and adjusted *P* values < 0.01. The criteria for the selection of significant DEPs in proteomics were a fold change of 1.5 and *P* values < 0.05. The data graphs of proteins expression levels in Western blot were created using GraphPad Prism software 6.0 (GraphPad Software, San Diego, CA, USA). The statistical significance was defined as *P* < 0.05 and the experiments were carried out at least three times.

## Results

### Identification of DEPs in CSCC tissue samples

**Fig. 2:**
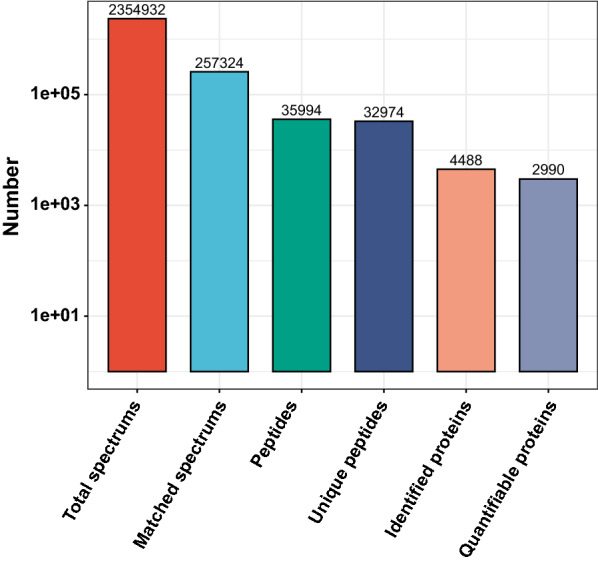
In total, 32,974 unique peptides were detected based on the MS/MS spectrum database search analysis, of which 4488 proteins were identified and 2990 proteins were quantified

**Fig. 3:**
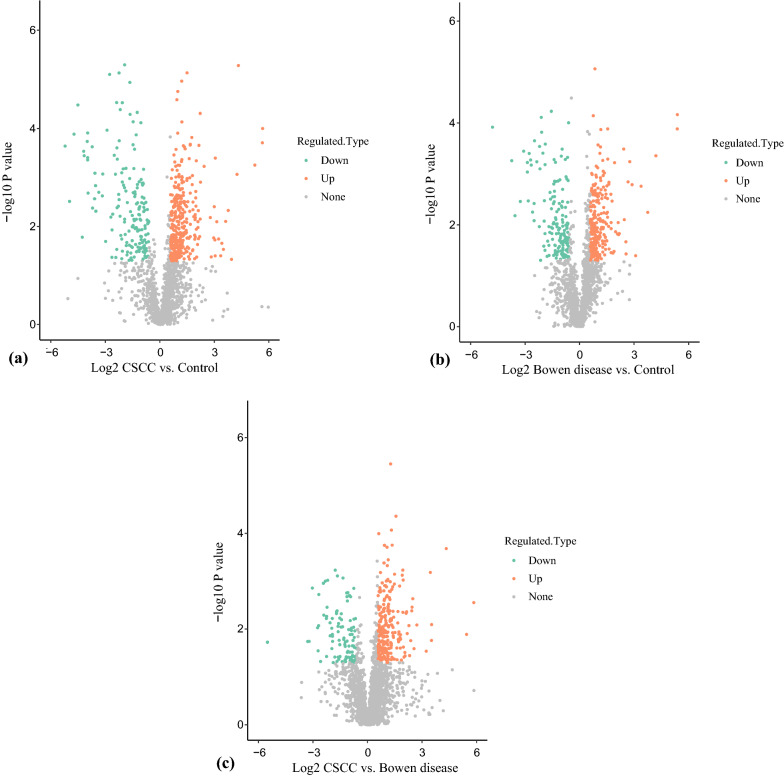
In the volcano plot, the red dot indicated a significantly upregulated protein and the blue dot indicated a significantly down regulated protein. The p-value was calculated using the two-sample t-test method. WhenP-value < 0.05, the difference expression amount changed by more than and less than 1.5 as a significantlyup-regulated and down-regulated change threshold, respectively. **a** When compared with healthy control, 332proteins were down-regulated and 169 proteins were up-regulated in CSCC. **b** In addition, a total of 246 proteinswere up-regulated and 154 proteins were down-regulated in the Bowen disease relative to the healthy control. **c** On the other hand, a total of 252 proteins were up-regulated and 104 proteins were down-regulated in the CSCCrelative to the Bowen disease

In our study, a total of 501 proteins were differentially expressed between the CSCC and healthy control, with 332 up-regulated and 169 down-regulated. In addition, a total of 252 proteins were up-regulated and 104 proteins were down-regulated in the CSCC relative to the Bowen disease. On the other hand, a total of 246 proteins were up-regulated and 154 proteins were down-regulated in the Bowen disease relative to the healthy control. The total spectra, matched spectra, peptides, unique peptides, and identified and quantified proteins obtained results are shown in Fig. 2. The volcano plot are shown in Fig. 3a–c.

### GO enrichment analysis

**Fig. 4:**
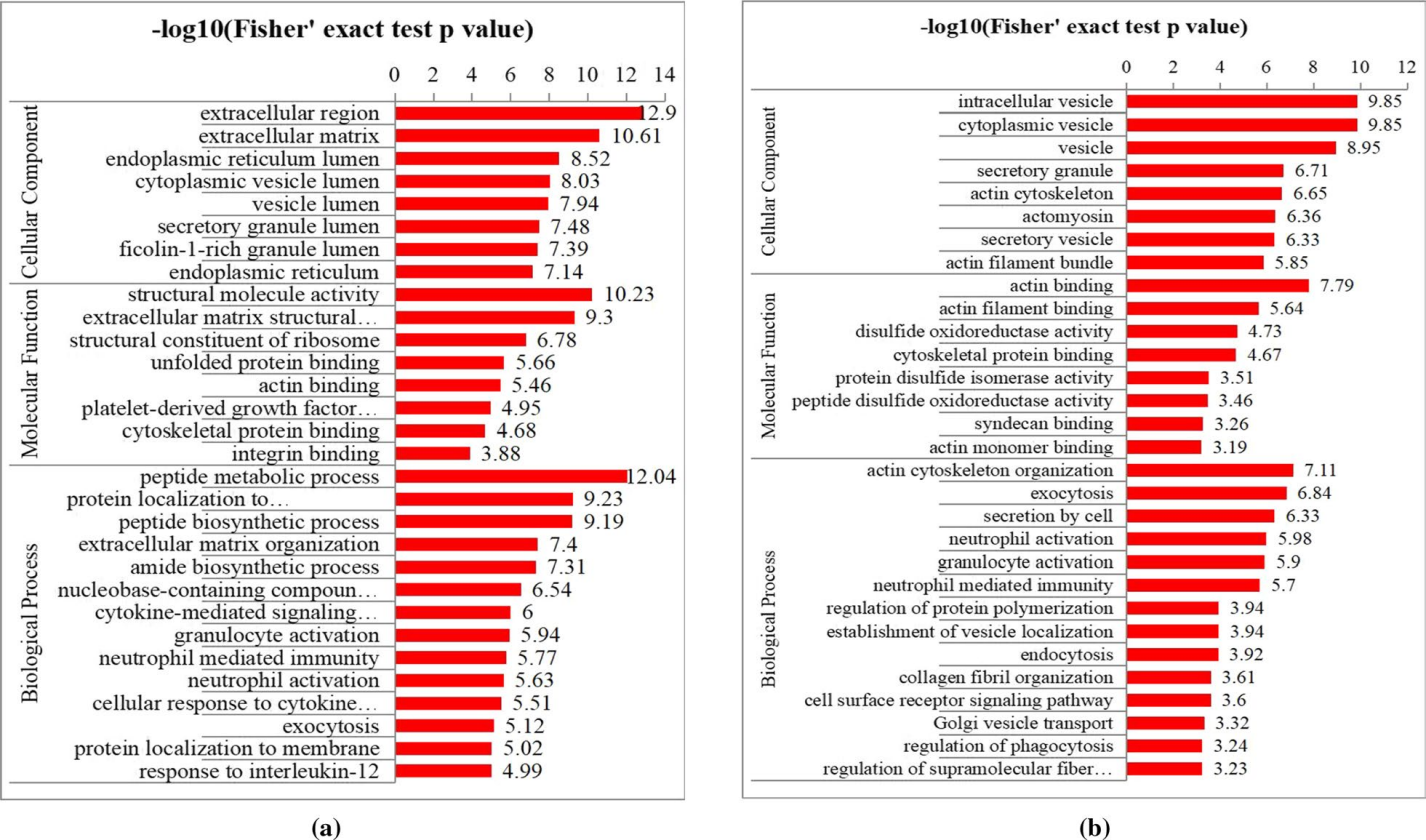
**a** A total of 501 differentially expressed proteins between CSCC and healthy control were categorized in 3 biological function types termed. **b** A total of 252 proteins were up-regulated and 104 proteins were down-regulated in the CSCC relative to the Bowen disease. The top 8 significantly enriched molecular function (MF) terms, 8 significantly enriched cellular component (CC) terms and 14 significantly enriched biological process (BP) terms are presented. The y-axis denotes the categories of GO terms

A total of 501 DEPs between CSCC and healthy control were found. GO annotation was made to categorize these DEPs based on 3 biological function types termed including molecular function (MF), cellular component (CC), and biological process (BP). The top 8 significantly enriched MF terms, 8 significantly enriched CC terms, and 14 significantly enriched BP terms are shown in Fig. 4a. For DEPs, the partial enriched MF terms included unfolded protein binding (*P* = 2.17*10^–6^), actin binding (*P* = 3.46*10^–6^) and platelet-derived growth factor binding (*P* = 1.13*10^–5^), which were related to protein binding process. The most enriched CC terms, such as extracellular region (*P* = 1.26*10^–13^), extracellular matrix (*P* = 2.47*10^–11^) and endoplasmic reticulum lumen (*P* = 2.99*10^–9^), mainly associated with the protein activation locations. As for BB terms, these DEPs were mainly enriched in nucleobase-containing compound catabolic process (*P* = 2.88*10^–7^), cytokine-mediated signaling pathway (*P* = 9.95*10^–7^), granulocyte activation (*P* = 1.14*10^–6^), neutrophil mediated immunity (*P* = 1.69*10^–6^), neutrophil activation (*P* = 2.33*10^–6^) and cellular response to cytokine stimulus (*P* = 3.09*10^–6^), which were related to immune, inflammation. On the other hand, when compared with Bowen disease, 356 DEPs in CSCC and were also categorized in MF, CC and BP terms. The 8 significantly enriched MF terms, 8 significantly enriched CC terms, and 14 significantly enriched BP terms are shown in Fig. 4b. The enriched MF terms, CC terms, and BP terms were similar to the results of Fig. 4a. For example, the partial common enriched MF terms (cytoplasmic vesicle lumen, vesicle lumen), CC terms (actin monomer binding, cytoskeletal protein binding) and BP terms (granulocyte activation, neutrophil mediated immunity, neutrophil activation) were found. Bowen disease, also called cutaneous squamous cell carcinoma in situ, without the invasion and metastasis features of malignant tumor. Importantly, 400 DEPs were found in Bowen disease relative to healthy control and these protein enriched multiple MF, CC and BP terms (Additional file 4: Figure. S1). As shown in Fig. 4, these enriched terms were not found apart from three BP terms (peptide metabolic process, peptide biosynthetic process amide biosynthetic process). Taken together, the results indicated that the enriched terms of Fig. 4 may associated with process of malignant tumors biological behavior.

### KEGG pathway enrichment analysis

**Fig. 5:**
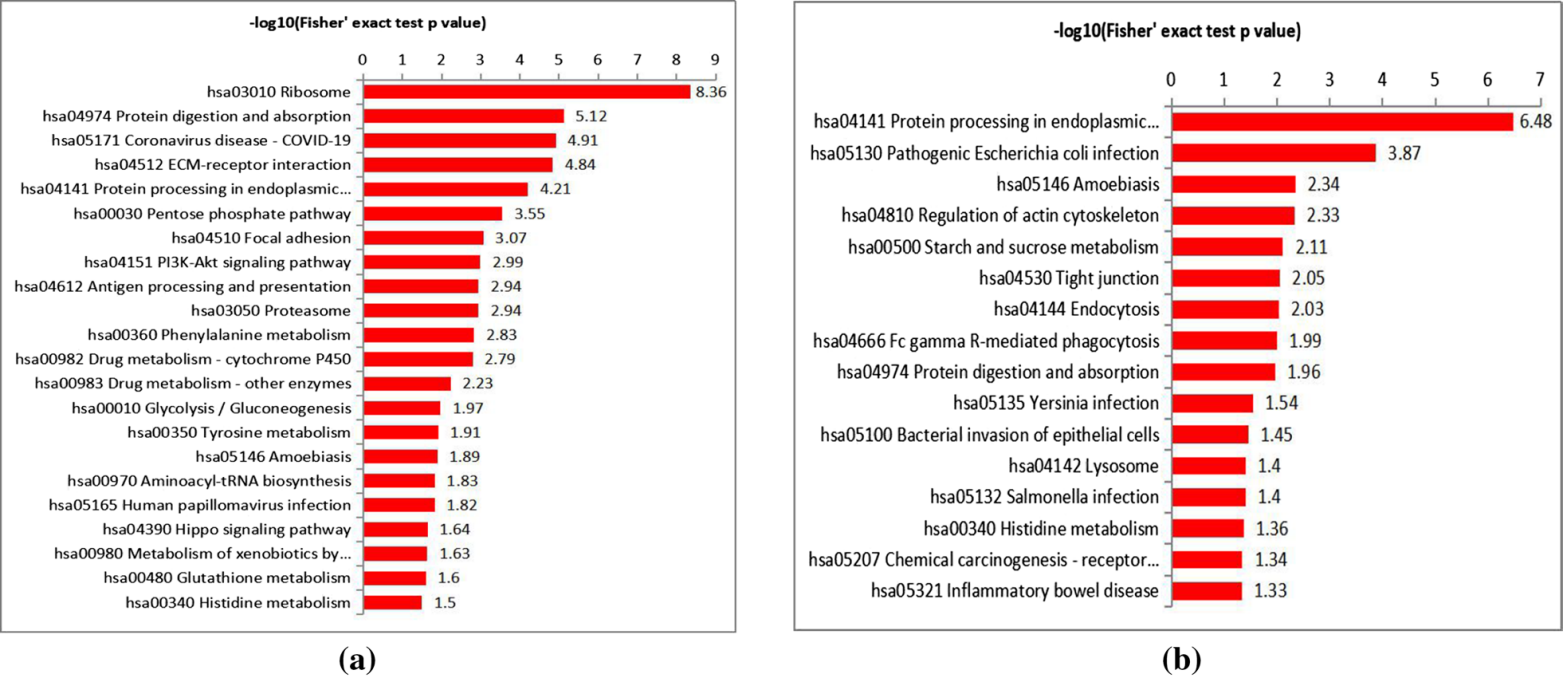
**a** KEGG pathways were generated based on the differentially expressed proteins between CSCC and healthy control. **b** KEGG pathways were generated based on the differentially expressed proteins between CSCC and Bowen disease

A total of 22 pathways, 16 pathways and 20 pathways were generated from the KEGG pathway analysis based on the proteins differentially expressed between the CSCC and healthy control, CSCC and Bowen disease, the Bowen disease and healthy control, respectively. When compare with healthy control, these DEPs in CSCC were most enriched in hsa03010, Ribosome (*P* = 4.39*10^–9^); hsa04974, Protein digestion and absorption (*P* = 7.64*10^–6^); hsa05171, Coronavirus disease-COVID-19 (*P* = 1.24*10^–5^); hsa04512, ECM-receptor interaction (*P* = 1.43*10^–5^); hsa04141, Protein processing in endoplasmic reticulum (*P* = 6.18*10^–5^); hsa00030, Pentose phosphate pathway (*P* < 0.001); hsa04510, Focal adhesion (*P* < 0.001); hsa04151, PI3K-Akt signaling pathway (*P* = 0.001) and others. In addition, 16 pathways were generated based on the DEPs between the CSCC and Bowen disease. The significantly enriched 8 KEGG pathways as follows: hsa04141, Protein processing in endoplasmic reticulum (*P* = 3.28*10^–7^); hsa05130, Pathogenic Escherichia coli infection (*P* < 0.001); hsa05146, Amoebiasis (*P* = 0.004); hsa04810, Regulation of actin cytoskeleton (*P* = 0.004); hsa00500, Starch and sucrose metabolism (*P* = 0.007); hsa04530, Tight junction (*P* = 0.008); hsa04144, Endocytosis (*P* = 0.009) and hsa04666, Fc gamma R-mediated phagocytosis (*P* = 0.010). The KEGG pathway analysis results are shown in Fig. 5 and Table 2. Besides, the DEPs among the Bowen disease and the healthy control which significantly enriched in 8 KEGG pathways are shown in Additional file 5: Figure S2 and Additional file 2: Table S2.

**Table 2:** The significantly enriched 8 KEGG pathways based on the differentially expressed proteins (CSCC vs. healthy control; CSCC vs. Bowen disease)

CSCC vs. healthy control			CSCC vs. Bowen disease		– log10(p value)
KEGG pathway	*P* value	– log10(p value)	KEGG pathway	*P* value	
hsa03010 Ribosome	4.39*10^–9^	8.36	hsa04141 Protein processing in endoplasmic reticulum	3.28*10^–7^	6.48
hsa04974 Protein digestion and absorption	7.64*10^–6^	5.12	hsa05130 Pathogenic Escherichia coli infection	< 0.001	3.87
hsa05171 Coronavirus disease -COVID-19	1.24*10^–5^	4.91	hsa05146 Amoebiasis	0.004	2.34
hsa04512 ECM-receptor interaction	1.43*10^–5^	4.84	hsa04810 Regulation of actin cytoskeleton	0.004	2.33
hsa04141 Protein processing in endoplasmic reticulum	6.18*10^–5^	4.21	hsa00500 Starch and sucrose metabolism	0.007	2.11
hsa00030 Pentose phosphate pathway	< 0.001	3.55	hsa04530 Tight junction	0.008	2.05
hsa04510 Focal adhesion	< 0.001	3.07	hsa04144 Endocytosis	0.009	2.03
hsa04151 PI3K-Akt signaling pathway	0.001	2.99	hsa04666 Fc gamma R-mediated phagocytosis	0.010	1.99

### PPI network of DEPs in CSCC

**Fig. 6:**
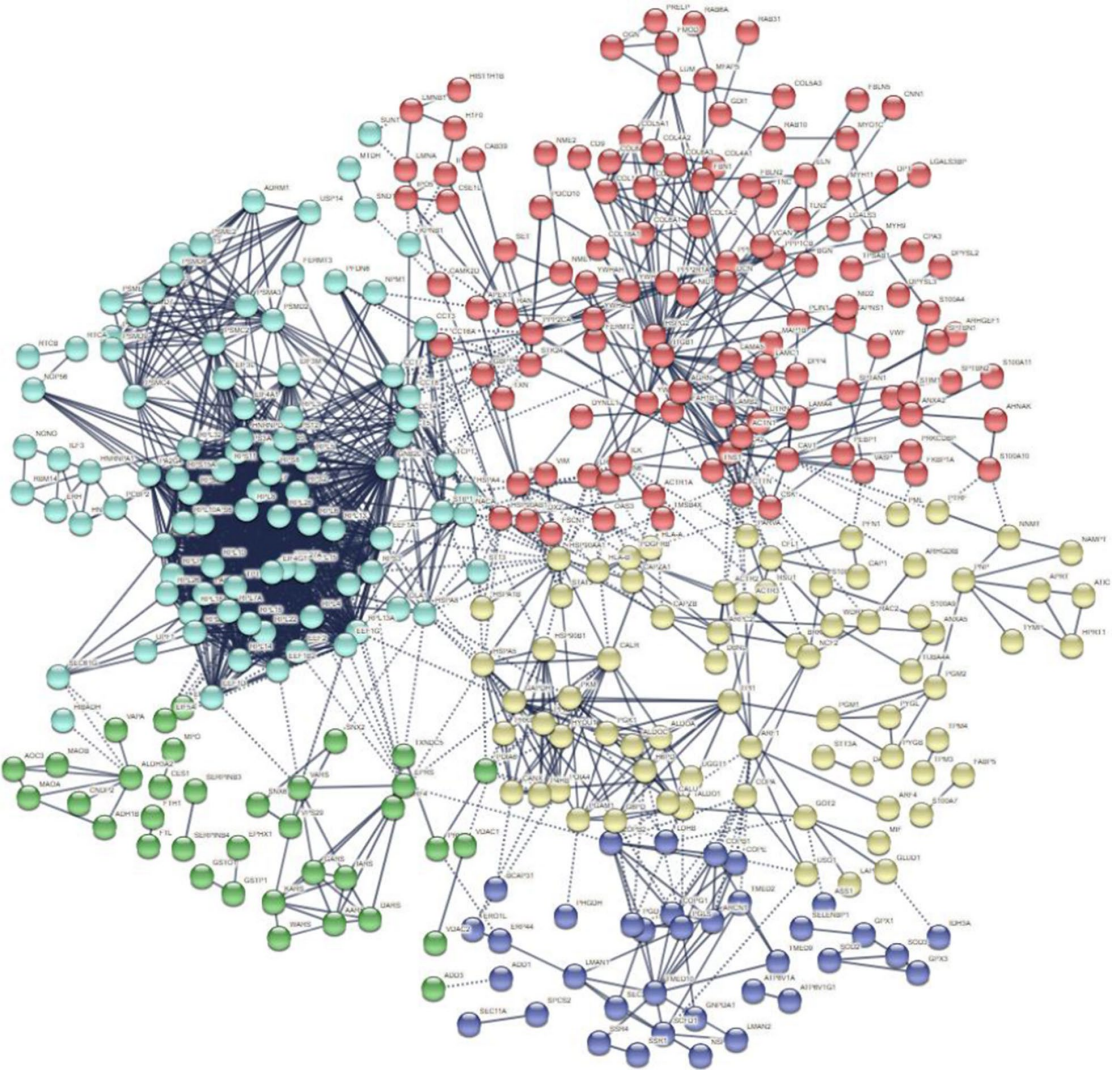
Protein–protein interaction (PPI) network of differentially expressed proteins and formed 5 clusters (clusters 1, red; clusters 2, yellow; clusters 3, green; clusters 4, light blue; clusters 5, navy blue) between CSCC and healthy control

To explore the interactions among these DEPs between CSCC and healthy control, we conducted a PPI network by using the STRING database and applied Cytoscape software to visualize the network. These DEPs had multiple interactions according to STRING predictions. In PPI network, the minimum required interaction score was set as more than 0.900 and disconnected nodes in the network were hided. A total of 5 clusters (clusters 1, red; clusters 2, yellow; clusters 3, green; clusters 4, light blue; clusters 5, navy blue) including 499 proteins were detected and showed in Fig. 6. The cluster 1 and cluster 4 accounted for most of the proteins (274/499, 54.91%), which suggested that these proteins have crucial roles in the pathogenesis of CSCC. The GO and KEGG enrichment analysis results of clusters 1 and 4 mainly involved with ECM-receptor interaction, protein digestion and absorption, focal adhesion, ribosome and others, this finding was mostly consistent with the results shown in Figs. 4 and 5. The protein node degrees indicated the interactions between two proteins. The higher degree indicated the closely interactions between two proteins.

### Data sources and crosstalk analysis

**Fig. 7:**
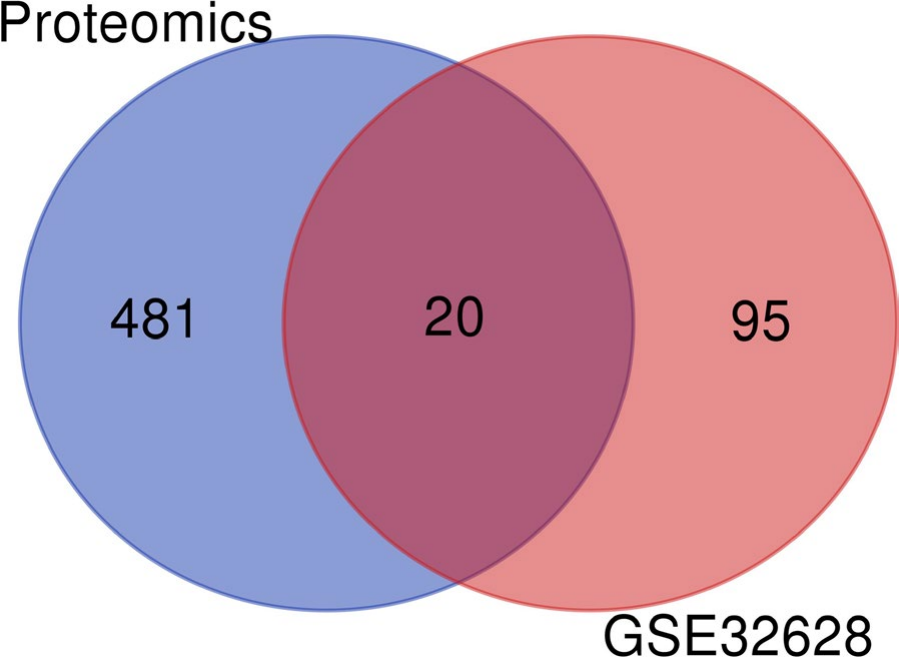
Venn diagram confirmed 20 gens that overlapped between the GSE32628 and our proteomics results

In GSE32628, adjusted *P* values < 0.01 and log_2_FC > 2 and (or) < -2 were set as cut-off criteria for screening out the differentially expressed gens in CSCC relative to normal skin tissues.When compared with normal skin tissues, a total of 115 differentially expressed gens (up-regulated: 91, down-regulated: 24) in CSCC were found. On the other hand, in our proteomics study, a total of 501 proteins were quantified, of which 332 were down-regulated and 169 were up-regulated in the CSCC relative to the healthy skin tissues. Finally, a Venn diagram was generated (Fig. 7) and 20 target gens (COL3A1, LUM, TNC, COL1A1, ALDH3A2, FSCN1, SERPINB4, SERPINB1, CD36, COL4A1, CSTB, GPX3, S100A7, ACTN1, SERPINB3, S100A8, RAB31, STAT1, SPRR1B, S100A9) that were overlapped between the GSE32628 and our proteomics results. Next, to explore the key gens which involved with the etiology of invasion and metastasis in CSCC, the significantly up- or down-regulated protein from these 20 gens in CSCC relative to Bowen disease were selected to verification. When compared with Bowen disease, the proteins expression values of TNC (degree: 1), FSCN1 (degree: 4), SERPINB1 (degree: 0), ACTN1 (degree: 5) and RAB31 (degree: 1) in CSCC were significantly up-regulated, while COL3A1 (degree: 9), COL1A1 (degree: 8), CD36 (degree:0) were significantly down-regulated. The expression levels of theses 8 proteins in our proteomics study between CSCC, Bowen disease and healthy control are shown in Table 3. Interestingly, in GSE32628, when compared with paired normal skin tissues, the mRNA expression values of TNC, FSCN1, SERPINB1, ACTN1, RAB31, COL3A1, COL1A1 and CD36 were all significantly increased in CSCC. The data was presented in Additional file 3: Table S3. The eight target proteins related biological functions or etiology in multiple tumors according to previous literature's reported [12–24] (partial) are shown in Table 4. On the other hand, the eight proteins involved the mainly KEGG pathways which those may associated with the etiology of CSCC development in our proteomics results are shown in Table 5.

**Table 3:** The differences of target proteins expression levels between CSCC, Bowen disease and healthy control

Protein accession	Gene name	CSCC vs. Control			Bowen disease vs. Control			CSCC vs. Bowen disease		
		Ratio	*P* values	up/down	Ratio	*P* values	up/down	Ratio	*P* values	up/down
P02461	COL3A1	0.064	< 0.001	down	0.310	0.025	down	0.208	0.006	down
P24821	TNC	4.372	0.018	up	1.297	0.255	–	3.371	0.017	up
P02452	COL1A1	0.056	< 0.001	down	0.259	0.008	down	0.214	0.003	down
Q16658	FSCN1	2.818	< 0.001	up	1.837	0.001	up	1.534	0.011	up
P30740	SERPINB1	20.065	< 0.001	up	3.573	0.036	up	5.615	0.002	up
P16671	CD36	0.065	0.002	down	0.343	0.203	–	0.189	0.005	down
P12814	ACTN1	1.547	0.019	up	0.987	0.889	–	1.567	0.001	up
Q13636	RAB31	2.910	0.003	up	1.530	0.344	–	1.902	0.014	up

The “-” indicated no significant differences

**Table 4:** The 8 target proteins related biological functions or etiology in multiple tumors according to previous literature's reported (partial)

Protein	Tumor	Regulated	Biological functions or etiology
COL1A1	Lung cancer [12]	Up	COL1A1 was correlated with immune infiltrating levels, including CD4^+^ T cells, macrophage, neutrophil, and dendritic cell in lung cancer
	Mesothelioma [13]	Up	COL1A1 expression was significantly correlated to the infiltration levels of CD4^+^ T cells, macrophages, and neutrophils in Mesothelioma
COL3A1	Ovarian carcinomas [14]	Up	COL3A1 higher expression was associated with shorter overall survival
CD36	Ovarian cancer [15]	Up	The omental adipocytes could reprogram tumour metabolism through the upregulation of CD36 in Ovarian cancer cells
TNC	Colorectal cancer [16]	Up	Higher expression of TNC was correlated with a poorer prognosis in stages II and III of CRC.
	Glioblastoma [17]	Up	Decreased TNC in the tumor microenvironment modulated behaviors of stromal cells, resulting in enlarged tumor blood vessels and activated microglia in tumors. Tenascin-C knockdown cells were sensitive to Temozolomide therapy
FSCN1	Renal cell carcinoma [18]	Up	Higher expression of FSCN1 led to an up-regulation of MMP9 and N-Cadherin in Renal cell carcinoma. Inhibition of PI3K/AKT or knockdown GSK-3βcould decreased the expression of FSCN1, and then attenuated Renal cell carcinoma invasion
	Adrenocortical carcinoma [19]	Up	FSCN1 higher expression was associated with worse prognoses; Higher expression of FSCN1 was associated with the tumour microenvironment and immune signatures in Adrenocortical carcinoma
ACTN1	Oral SCC [20]	Up	Inhibition of ACTN1 could induce cell cycle arrest, promote apoptosis, and inhibit EMT and cell proliferation, migration, and invasion in the OSCC cell lines
	Hepatocellular carcinoma [21]	Up	ACTN1 associated with Hippo signaling pathway activity and decreased Rho GTPases activities
RAB31	Pancreatic cancer [22]	Up	RAB31 higher expression was associated with shorter overall survival
	Cervical cancer [23]	Up	RAB31 knockdown inhibited the epithelial-mesenchymal transition and cytoskeletal rearrangement in cervical cancer cells. Overexpression of RAB31 inhibited MAPK6 degradation
SERPINB1	Glioma [24]	Down	SERPINB1 inhibited glioma migration and invasion probably by dampening the expression of matrix metalloproteinase-2

**Table 5:** The eight proteins involved the mainly KEGG pathways which those may associated with the etiology of CSCC development in our proteomics results

Protein	KEGG pathways
TNC	Focal adhesion; ECM-receptor interaction; Human papillomavirus infection; MicroRNAs in cancer; PI3K-Akt signaling pathway
FSCN1	MicroRNAs in cancer
SERPINB1	No enriched pathways
ACTN1	Focal adhesion; Viral carcinogenesis; Tight junction; Regulation of actin cytoskeleton; Adherens junction; Leukocyte transendothelial migration
RAB31	Endocytosis
COL3A1	Relaxin signaling pathway; Protein digestion and absorption; Platelet activation
COL1A1	Focal adhesion; Relaxin signaling pathway; Protein digestion and absorption; ECM-receptor interaction; Human papillomavirus infection; Proteoglycans in cancer; PI3K-Akt signaling pathway; Platelet activation
CD36	PPAR signaling pathway; Phagosome; ECM-receptor interaction; AMPK signaling pathway

### Western blot validation of TNC, FSCN1, SERPINB1, ACTN1, RAB31, COL3A1, COL1A1 and CD36

**Fig. 8:**
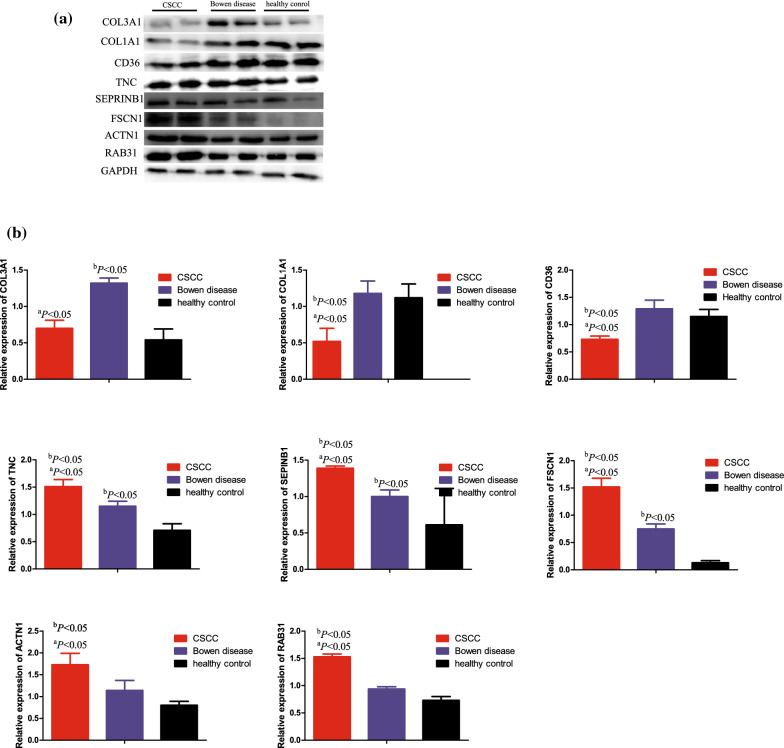
Western blotting validation. **a** Western blotting results of 8 proteins in CSCC, Bowen diseae and healthy control. **b** Western blotting quantitative analysis. Internal control was GAPDH. When compared with Bowen disease, a P < 0.05, When compared with healthy control, bP < 0.05

The Western blotting was carried out to verify the relative proteins expression levels of TNC, FSCN1, SERPINB1, ACTN1, RAB31, COL3A1, COL1A1 and CD36 in the independent verification samples (2 CSCC, 2 Bowen disease and 2 healthy skin tissues). According to the Western blotting analysis, when compared with the Bowen disease and healthy skin tissues, we found that the relative expression levels of TNC, FSCN1, SERPINB1, ACTN1 and RAB31 in the CSCC were significantly increased, while COL1A1 and CD36 were significantly decreased, and the differences were statistically significant (*P* < 0.05). Furthermore, the relative expression levels of TNC, FSCN1, SERPINB1 in the Bowen disease were also significantly increased, while the COL3A1 were also significantly decreased relative to the healthy control. The results are shown in Fig. 8.

### Silence of SERPINB1 inhibits migration and invasion of CSCC cell line A431

**Fig. 9:**
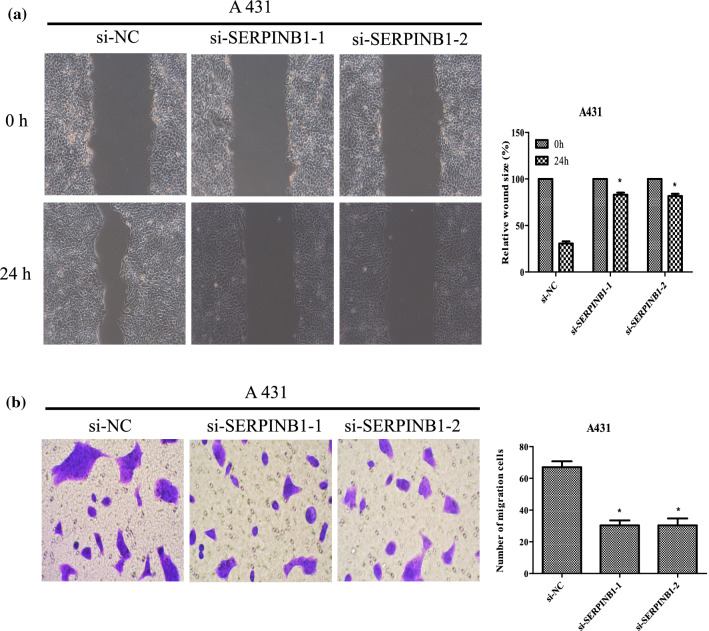
Knockdown of SERPINB1 suppressed the migration and invasion of A431 cells. **a** After transfection for 24 h, migration ability of A431 cells was determined by wound-healing assay, while **b** invasion ability of A431 cells were assessed by Transwell assays. Bars = 200 mm, *P < 0.01 compared with si-NC group

We first confirmed the efficiency of SERPINB1 knockdown, the expression of SERPINB1 mRNA and protein in CSCC cell line A431 were evaluated by qRT-PCR and western blotting, respectively. The SERPINB1 mRNA and protein expression levels in the cells transfected with si-SERPINB1 were decreased relative to the si-NC cells (Additional file 6: Figure S3c and S3d). To explore the migration and invasion ability of CSCC cell line A431 after SERPINB1 knockdown, wound healing assay and Trans-well assay were conducted. As shown in Fig. 9, the migration and invasion abilities of A431 cells were remarkable impaired (all *P* < 0.05). The results suggested that SERPINB1 knockdown could significantly inhibits CSCC cell migration and invasion.

## Discussions

CSCC, a keratinocyte-derived skin neoplasm with malignant potential, represents 20–50% of skin cancers and currently has an increasing incidence in the world [1]. The outcome of patients with CSCC mainly influenced by the TNM staging system, and the locally advanced, metastatic CSCC usually had poor outcomes. In recent years, the significantly progress has been made in the investigate the etiology of CSCC [25]. In spite of such advances, CSCC is often discovered at an advanced stage and the exact mechanisms of invasion, metastasis and or therapeutic targets are greatly needed. Proteins have a considerable number of biological functions within the human body. The quantitative proteomics analysis of FFPE samples technologies are sensitive and provide greater coverage of the tissue proteomes of many complicated diseases, especially in tumors [7, 26, 27]. Therefore, we compared the tumor samples proteomics change between CSCC (TNM staging: pT2 pN1 Mx, pT2 pN1 M1, pT1 pNx M1, pT3 pN1 M0 and pT3 pN1 Mx) and Bowen disease (primary CSCC), and to investigate the target proteins associated with mechanisms of invasion, metastasis in CSCC progress. In this proteomics study, we found that a numerous protein expression levels were significantly changed in the lesions between CSCC and Bowen disease. A total of 252 proteins were up-regulated and 104 proteins were down-regulated in the CSCC relative to the Bowen disease with a 1.5-fold change cut-off. Therefore, these significantly DEPs may play essential roles in the pathogenesis of malignant biological behaviors in CSCC. Bioinformatics tools were used to analyze the tumor biological behaviors and disease pathways which DEPs invloved. For example, our proteomics results indicated that the immune related inflammation (granulocyte activation, neutrophil mediated immunity, neutrophil activation, et al.), cell adhesion and tissue integrity (tight junction), devour process (endocytosis and Fc gamma R-mediated phagocytosis, et al*.*) from the results of Figs. 4, 5. Theses biological process were mainly associated with the pathogenesis of invasion, metastasis mechanisms in CSCC. On the other hand, a total of 501 proteins were differentially expressed between the CSCC and healthy control, with 332 up-regulated and 169 down-regulated. To verify the these DEPs/DEGs accurateness and relevance, we further compared our proteomics data with the transcriptomic data for 13 samples with CSCC and paired 13 normal skin tissues [28]. In two datasets, the expression levels of 20 common proteins trended in the same directions as in the RNA sequencing (RNA-seq) results (Fig. 7). Therefore, this 20 DEPs/DEGs were discerned to play an important role in the etiology of CSCC. And next, among 20 proteins, 8 proteins (TNC, FSCN1, SERPINB1, ACTN1, RAB31, COL3A1, COL1A1 and CD36) expressed levels showed significant differences between CSCC and Bowen disease.

The extensive family of COL gene products (collagens) is composed of several chain types, including fibril-forming interstitial collagens (types I, II, III and V) and basement membrane collagens (type IV), each type containing multiple isoforms. COL3A1 as well as COL1A1 play a role in cell adhesion process, important for maintaining normal tissue architecture and function [29]. We found the proteins expressed levels of COL3A1 and COL1A1 were significant decreased in CSCC relative to Bowen disease according to Western blot results. This results indicated the higher-regulation of COL3A1 and COL1A1 could suppressed CSCC progression, which may be involved in the etiology of altered cell adhesion process. Whereas, in contrast to CSCC, the higher expression of COL1A1 could promote lung cancer [12] and mesothelioma [13] progression via immune infiltration mechanisms (including CD4^+^ T cells, macrophage, neutrophil, and dendritic cell). Similar to COL1A1, higher expression of COL3A1 was associated with shorter overall survival in patients with ovarian carcinomas [14]. CD36 is a membrane glycoprotein on platelets, monocytes and umbilical vein endothelial cells. CD36 could binds to collagen and have an important functions in cell adhesion process [30]. In the previous reported [15], the CD36-driven omental adipocytes metabolic reprogramming and functions in tumor-associated immune cells lead to tumor immune tolerance and tumor development, especially in ovarian carcinomas. Moreover, CD36 mediates immunological recognition, inflammation, cell adhesion, and apoptosis in pathogenesis of cancer. In conflict with the previous findings, our study demonstrated that the CD36 serve as a tumor suppressor which was down-regulated in CSCC when compared with Bowen disease. Cell adhesion to extracellular matrix is an important physiological stimulus for organization of the actin-based cytoskeleton [31]. The tenascin family of extracellular matrix proteins includes Tenascin-C/R/X. Tenascin-C and Tenascin-X are expressed in several tissues during embryogenesis and in adult tissues undergoing active remodeling, such as tumors [32]. Higher expression of TNC was correlated with a poorer prognosis in stages II and III of colorectal cancer and could serve as a diagnostic biomarker [16]. Besides, the TNC may promote colorectal cancer progression and is involved in cancer stem cell-like properties via the Hedgehog signaling pathway [33]. In addition, Xia S, et al. [17] proved TNC knockdown cells were sensitive to Temozolomide therapy in glioblastoma. Our proteomics results also proved the TNC could driven the tumor progression in CSCC and this conclusion was consistent with the previous literature’s reported [34].

FSCN1, an actin-bindling protein, identifies dendritic cells in the blood and in tissues. ACTN1, one of the alpha actinins, which belong to cytoskeletal proteins and perform important biological functions, such as cell adhesion and migration. Our proteomics results showed expression of FSCN1 and ACTN1 were significant higher in CSCC relative to Bowen disease, and FSCN1 as well as ACTN1 may related to the proliferation, invasion and migration in CSCC progression process. In the previous reported, the higher-regulation of FSCN1 indicates worse prognosis for patients with renal cell carcinoma [18] and adrenocortical carcinoma [19]. Zhang M, et al. [18] indicated that higher expression of FSCN1 led to an up-regulation of MMP9 and N-Cadherin in renal cell carcinoma. Inhibition of PI3K/AKT or knockdown GSK-3b could decreased the expression of FSCN1, and then attenuated renal cell carcinoma invasion. On the other hand, Xie GF, et al. [20] indicated the inhibition of ACTN1 could induce cell cycle arrest, promote apoptosis, and inhibit cell proliferation, migration, and invasion in the oral squamous cell carcinoma cell lines.

RAB31 belongs to the Rab family, mainly localized in the trans-Golgi network and endosomes, which is the largest Ras superfamily of small molecule GTP binding proteins. Accumulating reports has indicated that RAB31 is widely involved in the prognosis of various cancers, including pancreatic cancer [22], cervical cancer [23] and others. Higher expression of RAB31 was significant associated with shorter overall survival in pancreatic cancer [22] and RAB31 knockdown could inhibited the epithelial-mesenchymal transition and cytoskeletal rearrangement in cervical cancer cells [23]. However, little is known regarding the RAB31 expression levels in CSCC. Our study confirmed that up-regulation of RAB31 in CSCC relative to Bowen disease and RAB31 may play a crucial role in CSCC invasion, metastasis process. SERPINB1 is a member of the Serpin family of protease inhibitors and associated with multiple cancers progression. Huasong G, et al. [24] suggested SERPINB1 could inhibited glioma migration and invasion probably by dampening the expression of matrix metalloproteinase-2. However, high expression of SERPINB1 in clinicopathologically invasive oral squamous cell carcinoma but not in normal oral mucosa (*P* < 0.01) was found by Tseng MY. et al. [35] Nevertheless, most study proved SERPINB1 may serve as a tumor suppressor and this conclusion was inconsistent with our results. Our study revealed that higher expression of SERPINB1 in CSCC relative to Bowen disease. This results may indicated the SERPINB1could as a novel therapeutic target to manage CSCC. In order to verify the effect of 8 proteins on the invasion and metastasis ability of CSCC and we selected the SERPINB1 for the next biomedical experiment. CSCC A431 cells were transfected with SERPINB1 small interfering RNA. After interfering with the expression of SERPINB1, the migration and invasion ability in the A431 cells were significantly decreased. This result indicated that inhibition of SERPINB1 can significantly inhibit the progression of CSCC. In addition, This result also demonstrated that 8 proteins may be related to the development of CSCC.

In conclusion, the proteomics technology provides a new method for the identification of key proteins associated with migration and invasion mechanisms in CSCC. These proteins were mainly involved in multiple pathways, including Focal adhesion, ECM-receptor interaction, Human papillomavirus infection, PI3K-Akt signaling pathway, PPAR signaling pathway, AMPK signaling pathway. Multiple protein factors, such as TNC, FSCN1, SERPINB1, ACTN1, RAB31, COL3A1, COL1A1 and CD36 could be as novel therapeutic target to manage CSCC. The most innovation of this manuscript was to explore the markers of CSCC progression using transcriptomics combined with proteomics methods in Chinese Han population. This also provides a theoretical basis for the targeted therapy of CSCC in the future.

### Limitations

The numbers of samples in proteomics are insufficient and a larger cohort could be of benefit however sourcing human samples is a challenge.

## Supplementary Information

**Additional file 1: Table S1.** The clinical characteristics of 6 enrolled individuals for independent verification.

**Additional file 2: Table S2.** The significantly enriched 8 KEGG pathways based on the differentially expressed proteins.

**Additional file 3: Table S3.** The TNC, FSCN1, SERPINB1, ACTN1, RAB31, COL3A1, COL1A1, and CD36 mRNA values in CSCC relative to healthy control in GSE32628.

**Additional file 4: Figure. S1.** a. A total of 246 proteins were up-regulated and 154 proteins were down-regulated in the Bowen disease relative to the healthy control. These differentially expressed proteins were categorized in 3 biological function types terms (MF, CC and BP). The top 8 significantly enriched molecular function (MF) terms, 8 significantly enriched cellular component (CC) terms and 14 significantly enriched biological process (BP) terms are presented. The y-axis denotes the categories of GO terms.

**Additional file 5: Figure S2**. KEGG pathways were generated based on the differentially expressed proteins between Bowen disease and healthy control.

**Additional file 6: Figure S3.** The SERPINB1 protein (a) and mRNA (b) expresison levels in CSCC cell line A431, SCL-1 and a human immortalized keratinocytes cell line Hacat were determined by Western blotting (a) and qRT–PCR (b).

**Additional file 7.** The raw data of 8 proteins in Western blot.

**Additional file 8.** The protocols for biological function experiments.

**Additional file 9.** The raw data of protomics of CSCC, Bowen disease and healthy control tissues.

## Data Availability

Due to the confidentiality of the original data, the full raw proteomics data in this study could be obtained by contacting the corresponding author T Yuan (Email: 857,923,284@qq.com). On the other hand, the full proteins/peptides and raw data of Western blot (eight markers) in this study was uploaded as supplementary materials.

## Abbreviations

CSCC: Cutaneous squamous cell carcinoma
FFPE: Formalin-fixed paraffin-embedded
