# Supplementary material for: From Bowen disease to cutaneous squamous cell carcinoma: eight markers were verified from transcriptomic and proteomic analyses
Source: J Transl Med. 2022 Sep 9;20:416. doi: 10.1186/s12967-022-03622-1 (PMC9462620; doi:10.1186/s12967-022-03622-1)
Supplement: Supplementary file 1 — Additional file 1: Table S1. The clinical characteristics of 6 enrolled individuals for independent verification. [file 12967_2022_3622_MOESM1_ESM.pdf]

Supplemental Table 1. The clinical characteristics of 6 enrolled individuals for independent verification.

Groups	Case	Age, years	Gender	Tissue locations	TNM staging
CSCC (n=2)	Case 1	67	Male	Cheek	pT2 pN1 M0
	Case 2	74	Male	Cheek	pT2 pN1 M1
Bowen disease (n=2)	Case 1	75	Male	Cheek	Primary CSCC
	Case 2	69	Female	Cheek	Primary CSCC
Healthy control (n=2)	Case 1	68	Male	Cheek	—
	Case 2	65	Female	Limbs	—
Statistics value	—	1.032	1.500	2.400	—
P value	—	0.456	0.472	0.301	—

Noted: The "—" indicated no value.
