# Supplementary material for: From Bowen disease to cutaneous squamous cell carcinoma: eight markers were verified from transcriptomic and proteomic analyses
Source: J Transl Med. 2022 Sep 9;20:416. doi: 10.1186/s12967-022-03622-1 (PMC9462620; doi:10.1186/s12967-022-03622-1)
Supplement: Supplementary file 2 — Additional file 2: Table S2. The significantly enriched 8 KEGG pathways based on the differentially expressed proteins. [file 12967_2022_3622_MOESM2_ESM.pdf]

Supplemental Table 2. The significantly enriched 8 KEGG pathways based on the differentially expressed proteins (Bowen disease vs. healthy control)

CSCC vs. healthy control		
KEGG pathway	*P* value	-log10(p value)
hsa04512 ECM-receptor interaction	$2.27*10^{-6}$	5.64
hsa04974 Protein digestion and absorption	$5.68*10^{-5}$	4.25
hsa04151 PI3K-Akt signaling pathway	<0.001	3.37
hsa05165 Human papillomavirus infection	<0.001	3.35
hsa03050 Proteasome	<0.001	3.35
hsa00360 Phenylalanine metabolism	<0.001	3.31
hsa04510 Focal adhesion	0.002	2.61
hsa00970 Aminoacyl-tRNA biosynthesis	0.003	2.46
