# Supplementary material for: From Bowen disease to cutaneous squamous cell carcinoma: eight markers were verified from transcriptomic and proteomic analyses
Source: J Transl Med. 2022 Sep 9;20:416. doi: 10.1186/s12967-022-03622-1 (PMC9462620; doi:10.1186/s12967-022-03622-1)
Supplement: Supplementary file 3 — Additional file 3: Table S3. The TNC, FSCN1, SERPINB1, ACTN1, RAB31, COL3A1, COL1A1, and CD36 mRNA values in CSCC relative to healthy control in GSE32628. [file 12967_2022_3622_MOESM3_ESM.pdf]

Supplemental Table 3. The TNC, FSCN1, SERPINB1, ACTN1, RAB31, COL3A1, COL1A1, and CD36 mRNA values in CSCC relative to healthy control in GSE32628.

ID	Gene	Adjusted *P* value	Log FC
ILMN_1719759	TNC	6.39E-10	2.38
ILMN_1808707	FSCN1	6.91E-11	2.19
ILMN_1769759	SERPINB1	2.02E-06	2.34
ILMN_1798892	ACTN1	4.32E-11	2.32
ILMN_1660691	RAB31	7.88E-12	2.18
ILMN_1773079	COL3A1	8.91E-07	2.03
ILMN_1701308	COL1A1	2.20E-09	2.84
ILMN_1784863	CD36	3.66E-06	2.06
