# Supplementary material for: From Bowen disease to cutaneous squamous cell carcinoma: eight markers were verified from transcriptomic and proteomic analyses
Source: J Transl Med. 2022 Sep 9;20:416. doi: 10.1186/s12967-022-03622-1 (PMC9462620; doi:10.1186/s12967-022-03622-1)
Supplement: Supplementary file 4 — Additional file 4: Figure. S1. a. A total of 246 proteins were up-regulated and 154 proteins were down-regulated in the Bowen disease relative to the healthy control. These differentially expressed proteins were categorized in 3 biological function types terms (MF, CC and BP). The top 8 significantly enriched molecular function (MF) terms, 8 significantly enriched cellular component (CC) terms and 14 significantly enriched biological process (BP) terms are presented. The y-axis denotes the categories of GO terms. [file 12967_2022_3622_MOESM4_ESM.pdf]

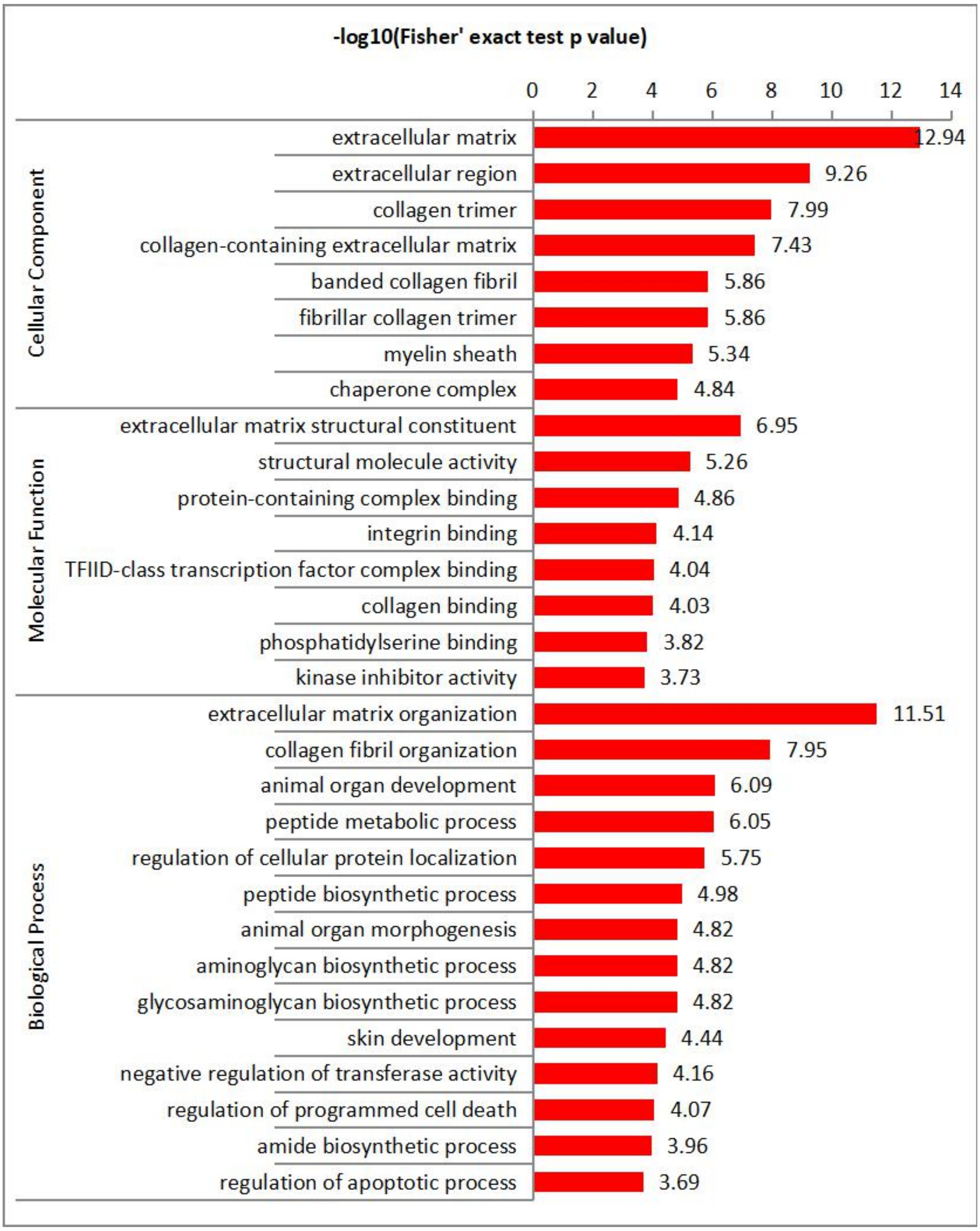

Supplemental Figure. 1. a. A total of 246 proteins were up-regulated and 154 proteins were down-regulated in the Bowen disease relative to the healthy control. These differentially expressed proteins were categorized in 3 biological function types terms (MF, CC and BP). The top 8 significantly enriched molecular function (MF) terms, 8 significantly enriched cellular component (CC) terms and 14 significantly enriched biological process (BP) terms are presented. The y-axis denotes the categories of GO terms.
