# Supplementary material for: From Bowen disease to cutaneous squamous cell carcinoma: eight markers were verified from transcriptomic and proteomic analyses
Source: J Transl Med. 2022 Sep 9;20:416. doi: 10.1186/s12967-022-03622-1 (PMC9462620; doi:10.1186/s12967-022-03622-1)
Supplement: Supplementary file 5 — Additional file 5: Figure S2. KEGG pathways were generated based on the differentially expressed proteins between Bowen disease and healthy control. [file 12967_2022_3622_MOESM5_ESM.pdf]

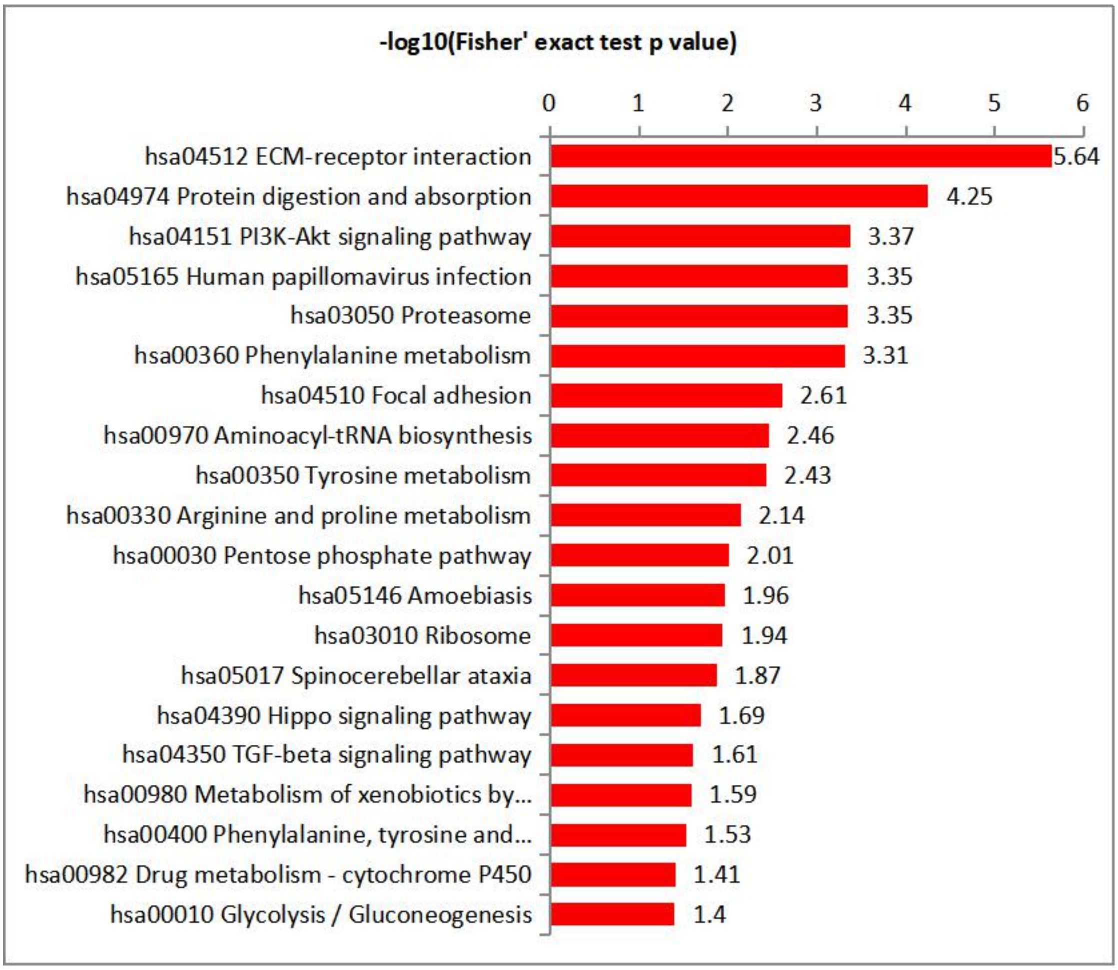

Supplemental Figure 2. KEGG pathways were generated based on the differentially expressed proteins between Bowen disease and healthy control.
