# Supplementary material for: From Bowen disease to cutaneous squamous cell carcinoma: eight markers were verified from transcriptomic and proteomic analyses
Source: J Transl Med. 2022 Sep 9;20:416. doi: 10.1186/s12967-022-03622-1 (PMC9462620; doi:10.1186/s12967-022-03622-1)
Supplement: Supplementary file 6 — Additional file 6: Figure S3. The SERPINB1 protein (a) and mRNA (b) expresison levels in CSCC cell line A431, SCL-1 and a human immortalized keratinocytes cell line Hacat were determined by Western blotting (a) and qRT–PCR (b). [file 12967_2022_3622_MOESM6_ESM.pdf]

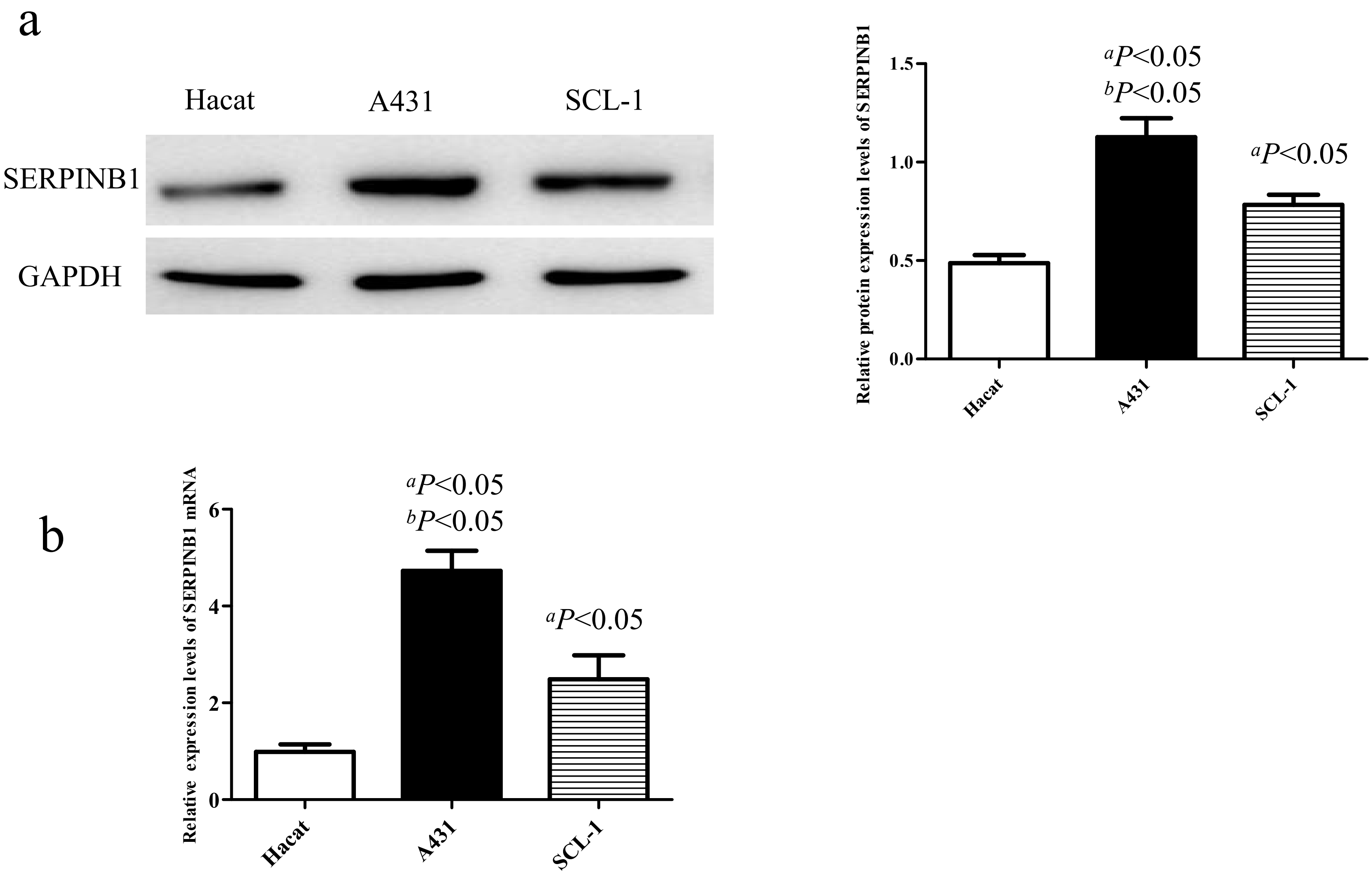
a
Hacat
A431
SCL-1
SERPINB1
GAPDH
Relative protein expression levels of SERPINB1
1.5
1.0
0.5
0.0
aP<0.05
bP<0.05
aP<0.05
Hacat
A431
SCL-1
b
Relative expression levels of SERPINB1 mRNA
6
4
2
0
aP<0.05
bP<0.05
aP<0.05
Hacat
A431
SCL-1

c

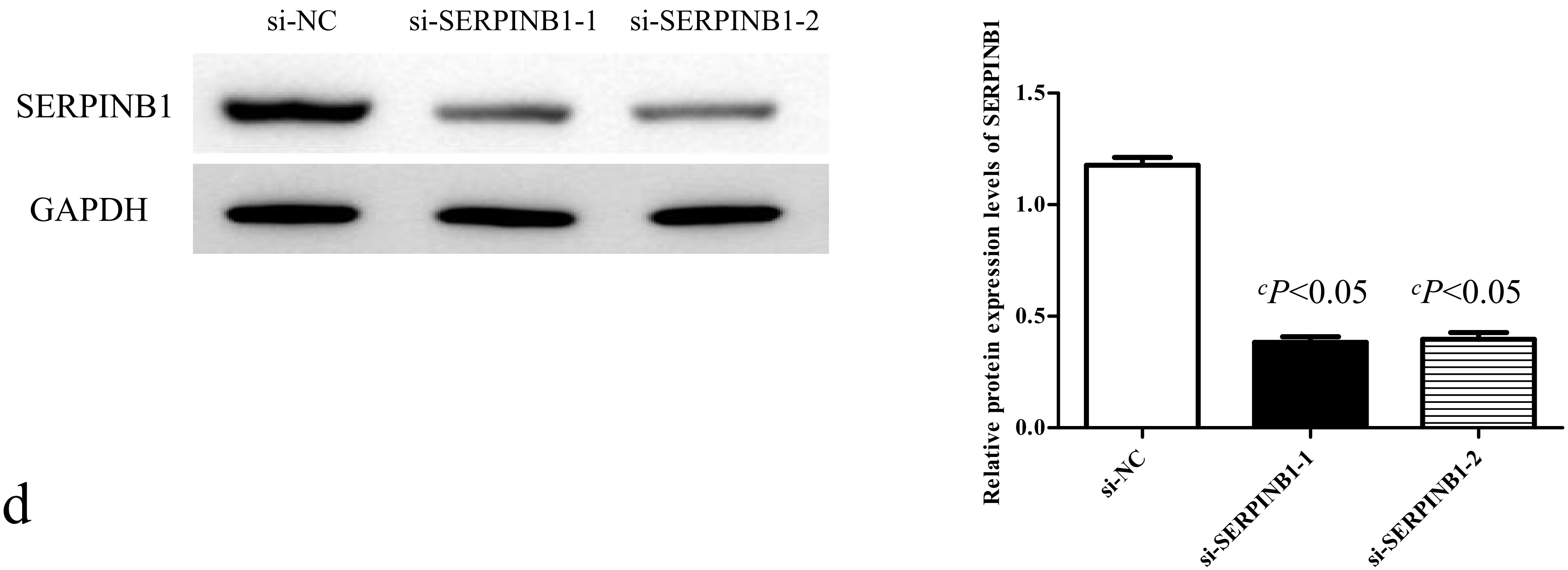
si-NC
si-SERPINB1-1
si-SERPINB1-2
SERPINB1
GAPDH
Relative protein expression levels of SERPINB1
1.5
1.0
0.5
0.0
cP<0.05
cP<0.05
si-NC
si-SERPINB1-1
si-SERPINB1-2

d

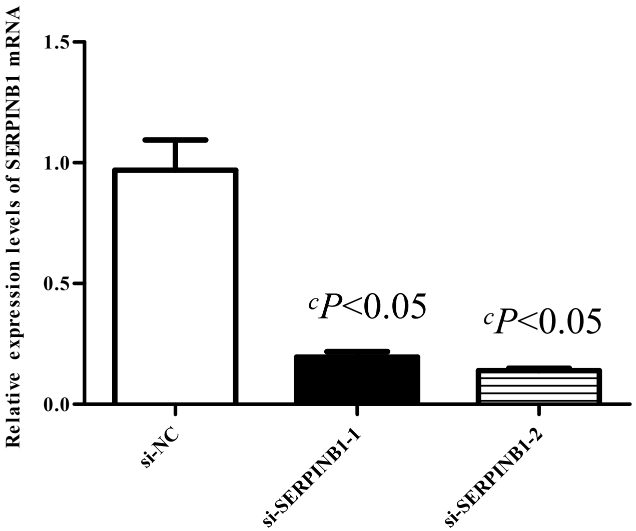
Relative expression levels of SERPINB1 mRNA
1.5
1.0
0.5
0.0
cP<0.05
cP<0.05
si-NC
si-SERPINB1-1
si-SERPINB1-2

Supplemental Figure 3. The SERPINB1 protein (a) and mRNA (b) expresison levels in CSCC cell line A431, SCL-1 and a human immortalized keratinocytes cell line Hacat were determined by Western blotting (a) and qRT–PCR (b). When compared with SCL-1, the SERPINB1 protein and mRNA expresison levels in A431 were significantly higher (P<0.05). Thus, the CSCC cell line A431 was selected for further research. When compared wtih si-NC, the SERPINB1 protein (c) and mRNA (d) expresison levels in si-SERPINB1-1 and si-SERPINB1-2 were significantly lower (P<0.05). When compared with Hacat, ^{a}P<0.05; When compared with SCL-1, ^{b}P<0.05; When compared with si-NC, ^{c}P<0.05. si-SERPINB1, A431 cells transfected with this specific siRNA; si-con, A431 cells transfected with nonspecific siRNA.
