# Supplementary material for: From Bowen disease to cutaneous squamous cell carcinoma: eight markers were verified from transcriptomic and proteomic analyses
Source: J Transl Med. 2022 Sep 9;20:416. doi: 10.1186/s12967-022-03622-1 (PMC9462620; doi:10.1186/s12967-022-03622-1)
Supplement: Supplementary file 7 — Additional file 7. The raw data of 8 proteins in Western blot. [file 12967_2022_3622_MOESM7_ESM.pdf]

		expression	relative expression	
	GAPDH			
	1	18524.104		
	2	19888.368		
	3	18927.953		
	4	18348.418		
	5	21198.296		
	6	25287.317		
	COL3A1			
CSCC	1	14450.518	0.780092684	0.78
	2	12492.69	0.628140529	0.63
BD	3	25840.761	1.365216883	1.37
	4	23270.782	1.268271848	1.27
Control	5	13558.347	0.639596079	0.64
	6	10953.468	0.433160544	0.43
	COL2A1			
CSCC	1	12124.468	0.654523857	0.65
	2	7826.447	0.393518815	0.39
BD	3	20035.246	1.058500409	1.06
	4	23781.589	1.296111142	1.30
Control	5	26650.66	1.257207655	1.26
	6	24891.589	0.984350732	0.98
	CD36			
CSCC	1	11603.589	0.626404872	0.63
	2	16413.347	0.825273698	0.83
BD	3	22277.125	1.17694317	1.18
	4	25811.953	1.406767221	1.41
Control	5	24156.489	1.139548622	1.34
	6	24207.075	0.957281273	0.96
	TNC			
CSCC	1	29638.489	1.599995822	1.60
	2	28032.539	1.409494183	1.41
BD	3	20674.104	1.092252501	1.09
	4	22377.903	1.219609396	1.22
Control	5	16945.125	0.7993626	0.80
	6	15858.225	0.627121691	0.63
	SEPRINB1			
CSCC	1	26058.64	1.406742264	1.41
	2	27215.468	1.368411325	1.37
BD	3	20320.368	1.073563951	1.07
	4	17196.368	0.937212571	0.94
Control	5	20508.853	0.96747649	0.97
	6	6541.418	0.25868375	0.26
	FSCN1			
CSCC	1	30146.539	1.627422249	1.63
	2	27929.368	1.404306678	1.40

BD	3	15496.418	0.818705435	0.82
	4	12623.953	0.688013157	0.69
Control	5	3339.326	0.157528039	0.16
	6	2378.669	0.094065693	0.09
	ACTN1			
CSCC	1	35449.075	1.913672856	1.91
	2	30775.468	1.547410426	1.55
BD	3	18415.104	0.972905205	0.97
	4	23943.489	1.304934791	1.30
Control	5	18450.468	0.870375053	0.87
	6	18703.761	0.739649881	0.73
	RAB31			
CSCC	1	27699.711	1.495333378	1.50
	2	31015.539	1.559481351	1.56
BD	3	17248.347	0.911263199	0.91
	4	17747.761	0.967263826	0.97
Control	5	16503.882	0.778547578	0.78
	6	17097.033	0.676110993	0.68

0.704116606	0.70	MEAN
0.107446399	0.11	STD
1.316744365	1.32	MEAN
0.068550492	0.07	STD
0.536378312	0.54	MEAN
0.145971966	0.15	STD
0.524021336	0.52	MEAN
0.184558435	0.18	STD
1.177305775	1.18	MEAN
0.16801616	0.17	STD
1.120779193	1.12	MEAN
0.19293898	0.19	STD
0.729823003	0.73	MEAN
0.134987667	0.06	STD
1.291855195	1.29	MEAN
0.162510145	0.16	STD
1.148414947	1.15	MEAN
0.270303835	0.13	STD
1.505	1.51	MEAN
0.134350288	0.13	STD
1.155	1.15	MEAN
0.091923882	0.09	STD
0.713242145	0.71	MEAN
0.121792714	0.12	STD
1.388371132	1.39	MEAN
0.025980704	0.03	STD
1.003606285	1.00	MEAN
0.093894892	0.09	STD
0.61308012	0.61	MEAN
0.501192152	0.50	STD
1.515864464	1.52	MEAN
0.157766533	0.16	STD

0.753359296	0.75	MEAN
0.092413396	0.09	STD
0.125796866	0.13	MEAN
0.044874655	0.04	STD
1.730541641	1.73	MEAN
0.258986648	0.26	STD
1.138919998	1.14	MEAN
0.234780371	0.23	STD
0.8	0.80	MEAN
0.098994949	0.09	STD
1.527407364	1.53	MEAN
0.045359467	0.05	STD
0.939263513	0.94	MEAN
0.039598423	0.04	STD
0.727329285	0.73	MEAN
0.072433604	0.07	STD
