# Supplementary material for: From Bowen disease to cutaneous squamous cell carcinoma: eight markers were verified from transcriptomic and proteomic analyses
Source: J Transl Med. 2022 Sep 9;20:416. doi: 10.1186/s12967-022-03622-1 (PMC9462620; doi:10.1186/s12967-022-03622-1)
Supplement: Supplementary file 8 — Additional file 8. The protocols for biological function experiments. [file 12967_2022_3622_MOESM8_ESM.pdf]

**RT-qPCR assay:** The total RNA from A431 cell line were extracted by TRIzol reagent (Invitrogen). The RNA were reverse-transcribed into complementary DNA by PrimeScript® RT reagent Kit (Takara Bio Inc., Kusatsu, Shiga, Japan) on a S1000TM Thermal Cycler (BioRad Laboratories, Inc., Hercules, CA, USA) at 37 °C for 10 minutes and 85 °C for 6 seconds. The SERPINB1 mRNA level was measured using the SYBR® Premix Ex Taq™ (Takara Bio Inc.,) on a QuantStudio™ 6 Flex Real-Time PCR System (Thermo Fisher Scientific, Inc.). The initial denaturation was performed at 90°C for 30 s, and the subsequent PCR reaction was conducted at 90°C for 5 s and at 60°C for 30 s for 40 cycles. The primers of SERPINB1 and GAPDH were as follows: SERPINB1, Forward: GCCAAGGTCCTGGAAATACCA, Reverse: TTCCATCAATTTCTCAGCAGTGA; GAPDH, Forward: CTGGCCGGGACCTGACT, Reverse: GCAGCCGTGGCCATCTC. The experiment were analyzed in triplicate. The relative fold changes were calculated by the $2^{-\triangle\triangle Ct}$ method, and GAPDH was used as an internal reference.

**Western Blot assay:** The cells were lysed in RIPA lysis buffer (Beyotime Biotechnology, Inc., Jiangsu, China) with PMSF (1 mmol/L) (Beyotime Biotechnology, Inc., Jiangsu, China) for 10 minutes on ice with intermittent vortex, followed by centrifuging at 12,000 g at 4°C for 15 minutes. The supernatants were collected immediately. The protein concentrations were analyzed using BCA Protein Assay kit (Beyotime Biotechnology, Inc., Jiangsu, China). Protein lysates separated by sulfate-polyacrylamide gel electrophoresis were then transferred onto polyvinylidene difluoride membrane (Millipore, MA, USA). The anti-SERPINB1 antibody ( Abcam, Inc., USA, catalogue number: Ab181084, with antibody dilutions of 1: 2000) was added as primary antibody and anti-GAPDH was used as an internal reference control. The bands were detected by enhanced chemiluminescence using PierceTM SuperSignal West Femto Chemiluminescent substrate (Thermo Fisher Scientific, Inc.). The experiment were analyzed in triplicate.

**Wound healing assay:** Cells were seeded into six-well plates and then cultured for 24 h to 90% confluence. A linear wound was performed by scratching the sub-confluent cell monolayer using a 200 μl pipette tip, and the debris was removed by washing with phosphate-buffered saline. After incubation at 37 °C for 48 h, cells were photographed and wound width were obtained at 0 h and 24 h. The experiment were analyzed in triplicate.

**Trans-well assays:** The cells were harvested and re-suspended in 200 μl serum-free medium at a density of $10^6$/mL. The re-suspended cells were seeded into the upper chamber of 24-well trans-well chambers with Matrigel (BD Biosciences, NJ, USA) for the invasion assay. About 600 μl 20% fetal bovine serum medium was added to the lower chamber. After 24 h, cells on the lower surface of the inserts were stained with 0.1% crystal violet. Cells were viewed under a microscope and counted in five random microscopic fields.The experiment were analyzed in triplicate.
